# Epigallocatechin gallate affects glucose metabolism and increases fitness and lifespan in *Drosophila melanogaster*

**DOI:** 10.18632/oncotarget.5215

**Published:** 2015-09-08

**Authors:** Anika E. Wagner, Stefanie Piegholdt, Doerte Rabe, Nieves Baenas, Anke Schloesser, Manfred Eggersdorfer, Achim Stocker, Gerald Rimbach

**Affiliations:** ^1^ Institute of Human Nutrition and Food Science, University of Kiel, Kiel, Germany; ^2^ Centro de Edafología y Biología Aplicada del Segura (CEBAS-CSIC), Campus Universitario de Espinardo, Murcia, Spain; ^3^ DSM Nutritional Products, Basel, Switzerland; ^4^ Department of Chemistry and Biochemistry, University of Bern, Bern, Switzerland

**Keywords:** Gerotarget, epigallocathechin-3-gallate, green tea, life span, Drosophila melanogaster

## Abstract

In this study, we tested whether a standardized epigallocatechin-3-gallate (EGCG) rich green tea extract (comprising > 90% EGCG) affects fitness and lifespan as well as parameters of glucose metabolism and energy homeostasis in the fruit fly, *Drosophila melanogaster*. Following the application of the green tea extract a significant increase in the mean lifespan (+ 3.3 days) and the 50% survival (+ 4.3 days) as well as improved fitness was detected. These effects went along an increased expression of Spargel, the homolog of mammalian PGC1α, which has been reported to affect lifespan in flies. Intriguingly, in flies, treatment with the green tea extract decreased glucose concentrations, which were accompanied by an inhibition of α-amylase and α-glucosidase activity. Computational docking analysis proved the potential of EGCG to dock into the substrate binding pocket of α-amylase and to a greater extent into α-glucosidase. Furthermore, we demonstrate that EGCG downregulates insulin-like peptide 5 and phosphoenolpyruvate carboxykinase, major regulators of glucose metabolism, as well as the *Drosophila* homolog of leptin, unpaired 2. We propose that a decrease in glucose metabolism in connection with an upregulated expression of Spargel contribute to the better fitness and the extended lifespan in EGCG-treated flies.

## INTRODUCTION

The consumption of green tea (*Camellia sinensis*) has been associated with various health benefits [1–4]. The leaves of green tea offer a wide spectrum of different phytochemicals that may vary according to environmental conditions and processing procedures. Approximately 30% of the green tea dry weight comes from polyphenols of which 60–80% are catechins [5]. Catechins and in particular epigallocatechin-3-gallate (EGCG), which accounts for up to 80% of the catechins, are suggested to mediate the health-promoting effects of green tea [6–8].

In fact, studies in various model organisms indicate a lifespan extension by green tea and EGCG treatment. This has been shown in both invertebrates and in mammalian species [9–13]. However, the underlying cellular and molecular mechanisms are not clear. Bartholome and colleagues observed higher levels of the FoxO ortholog DAF-16, which increased the expression of its target gene Sod-3, and extended lifespan in *Caenorhabditis elegans* [14]. In *Drosophila melanogaster*, EGCG extended lifespan *via* an induction of endogenous antioxidant enzymes [12]. Additionally, in mice receiving tea polyphenols from the 13^th^ month of life onward, lifespan was significantly longer than in the corresponding control animals [9]. Furthermore, rats treated with EGCG also exhibited a significantly longer lifespan that was accompanied by a decrease in inflammation and oxidative stress as well as an increase of FOXO3a and SIRT1, both centrally involved in the regulation of longevity [10]. It has been demonstrated that aging is associated with a decrease in mitochondrial biogenesis and in consequence with a loss in the expression of PPARγ co-activator α (PGC1α), the master switch of energy metabolism [15, 16]. Interestingly, green tea polyphenols have been shown to induce both PGC1α mRNA and protein expression in male Sprague-Dawley rats [17].

The aim of the present study was to elucidate the underlying mechanisms of the lifespan extending effect of a caffeine-free green tea extract containing more than 90% EGCG in *Drosophila melanogaster*. We analyzed the regulatory effects of EGCG on parameters involved in energy homeostasis and glucose metabolism. In addition, fitness, as marker for an improved health span, was assessed.

## RESULTS

### EGCG extends lifespan and improves fitness in *Drosophila melanogaster*

**Figure 1:**
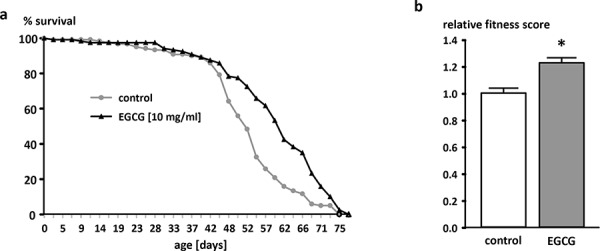
Effect of EGCG (10 mg/ml) supplementation on lifespan in male W^1118^ *Drosophila melanogaster* **a.** One representative experiment out of two is shown. *n* = 120 flies per group, *p* < 0.05 log-rank test. **b.** Relative fitness levels of male W^1118^ *Drosophila melanogaster* reared on an EGCG (10 mg/ml)-supplemented diet for 30 days. The data are expressed as the mean + SEM (*n* = 100). * indicates significant differences compared to the control group (*p* < 0.05, Student's *t*-test).

The application of 10 mg/ml EGCG increased the mean lifespan (+ 3.3 days) and the 50% survival (+ 4.3 days) compared to control flies (Figure 1a). Flies reared on EGCG-supplemented medium for 30 days exhibited a significantly higher fitness level in the climbing assay than the control flies (Figure 1b).

### EGCG inhibits α-amylase and α-glucosidase *in vitro* and *in vivo* and decreases glucose levels in *Drosophila melanogaster*

**Figure 2:**
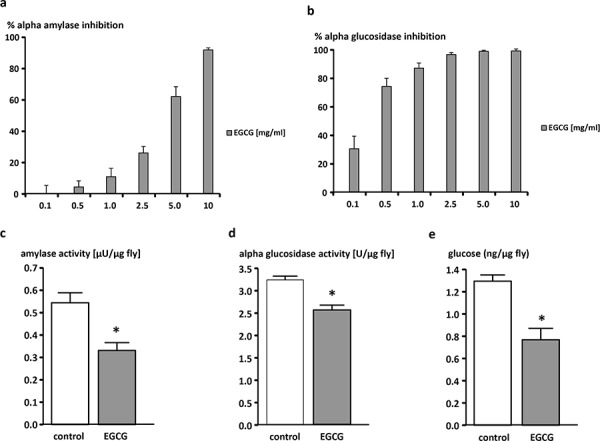
EGCG-dependent inhibition of α-amylase and α-glucosidase activity *in vitro* (a, b) and *in vivo* (c, d) and decrease of glucose levels *in vivo* (e) Dose-dependent inhibition of **a.** α-amylase and **b.** α-glucosidase by EGCG *in vitro*. The data are expressed as the mean + SEM (*n* = 3). **c.** α-amylase activity, **d.** α-glucosidase activity, and **e.** glucose levels of male W^1118^ *Drosophila melanogaster* relative to fly weights. Flies were reared on an EGCG (10 mg/ml)-supplemented or control diet for 10 days. The data are expressed as the mean + SEM (*n* = 5–6, indicating extraction from 5–6 × 5 flies). * indicates significant differences in EGCG-treated flies compared to flies fed control medium for 10 days (*p* < 0.05, Student's t-test).

EGCG dose-dependently inhibited both α-amylase and α-glucosidase *in vitro* (Figure 2a, 2b). Accordingly, flies reared for 10 days on the EGCG-supplemented diet showed significantly lower levels of both amylase (Figure 2c) and α-glucosidase activity (Figure 2d). As shown in Figure 2e, glucose levels were lowered by 25% following EGCG-treatment.

### Molecular docking of EGCG on human salivary α-amylase

**Figure 3:**
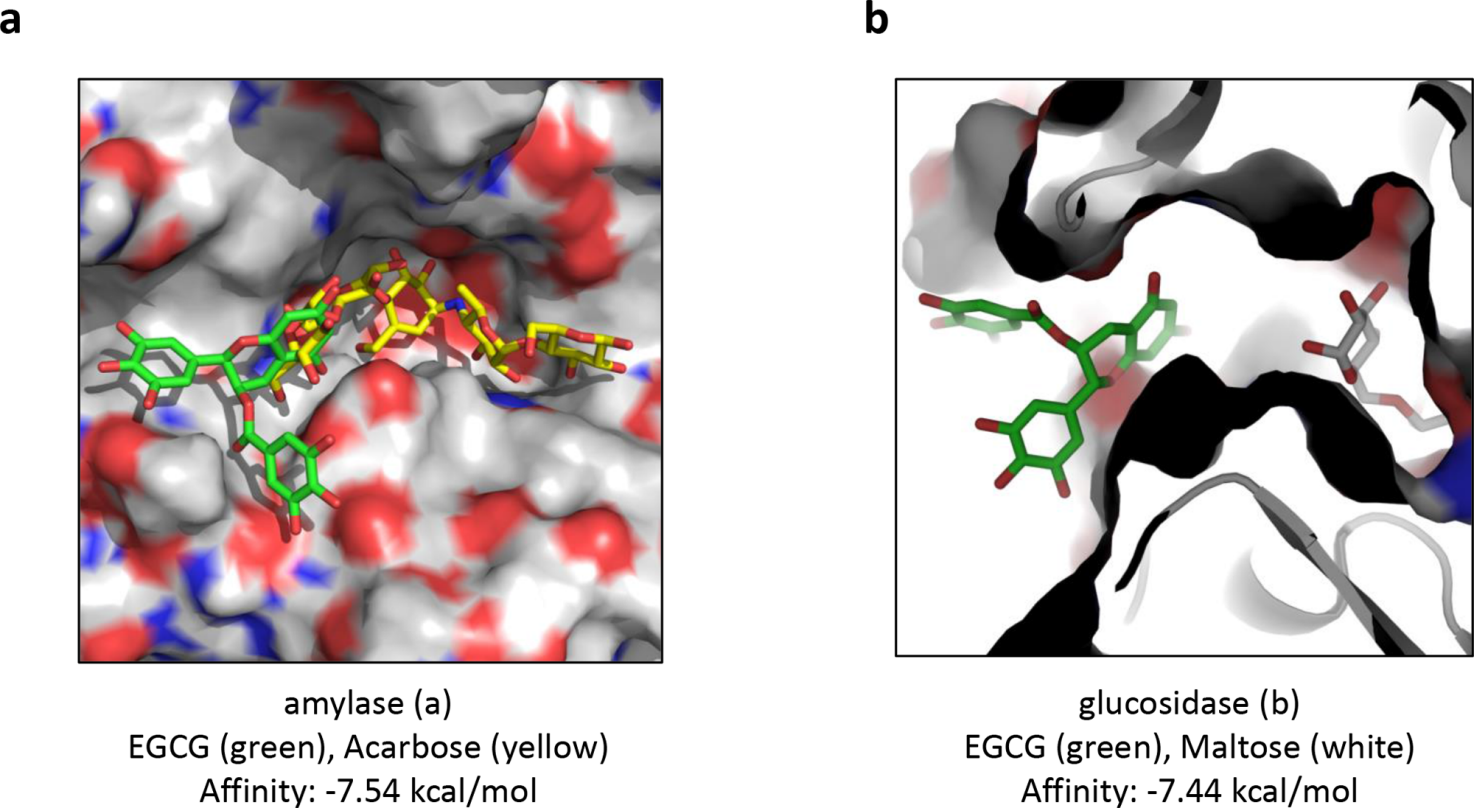
Predicted binding modes of EGCG docked with the X-ray structure of human salivary α-amylase and with the homology model of α-glucosidase from baker's yeast **a.** The interaction between EGCG and human salivary α-amylase. Illustration of the surface of the substrate binding cleft with bound EGCG (green sticks) and the 3D-superposed acarbose (yellow sticks) from pancreatic α-amylase to visualize pseudo-substrate position (yellow) within the ligand binding cleft relative to docked EGCG inhibitor (green). **b.** The interaction between EGCG and α-glucosidase from baker's yeast. Illustration of the surface of the substrate binding cavity bound to its competitive inhibitor maltose (grey sticks) relative to docked EGCG (green sticks). Images were depicted using the Pymol software.

In order to gain insight into the putative binding mode of EGCG with human salivary α-amylase blind docking for EGCG with the crystallographic model of apo-amylase was carried out [27]. Figure 3a illustrates a predicted binding mode of EGCG snugly fitting into the left arm of the polysaccharide-binding cleft of human salivary amylase. The binding free energies of a cluster of eight binding poses were averaged (−7.29 kcal/mol ± SEM = 0.056 kcal/mol) to estimate the affinity from the predicted binding intensities of the docking interaction of EGCG at the active site of human salivary α-amylase. The 3D-superposition of the pig amylase:acarbose inhibitor-complex with the *in silico* model of human salivary α-amylase:EGCG complex produced steric clashes of the inhibitor with EGCG in close proximity to the active site.

### α-Glucosidase homology model preparation and molecular docking of EGCG

Using the web-based SWISS-MODEL service dedicated to protein structure homology modeling [29] a α-glucosidase homology model was built. Blind docking for EGCG with the homology model of α-glucosidase was carried out using the web-based SwissDock service [27]. Figure 3b illustrates the predicted binding mode of EGCG plugging the entry of the substrate-binding cavity of the α-glucosidase. The binding free energies of a cluster of eight binding poses were averaged (−7.44 kcal/mol ± SEM = 0.001 kcal/mol) to estimate the affinity from the predicted binding intensities of the docking interaction of EGCG at the entry of the binding pocket of α-glucosidase.

### EGCG affects the expression of biomarkers related to energy homeostasis and energy metabolism

**Figure 4:**
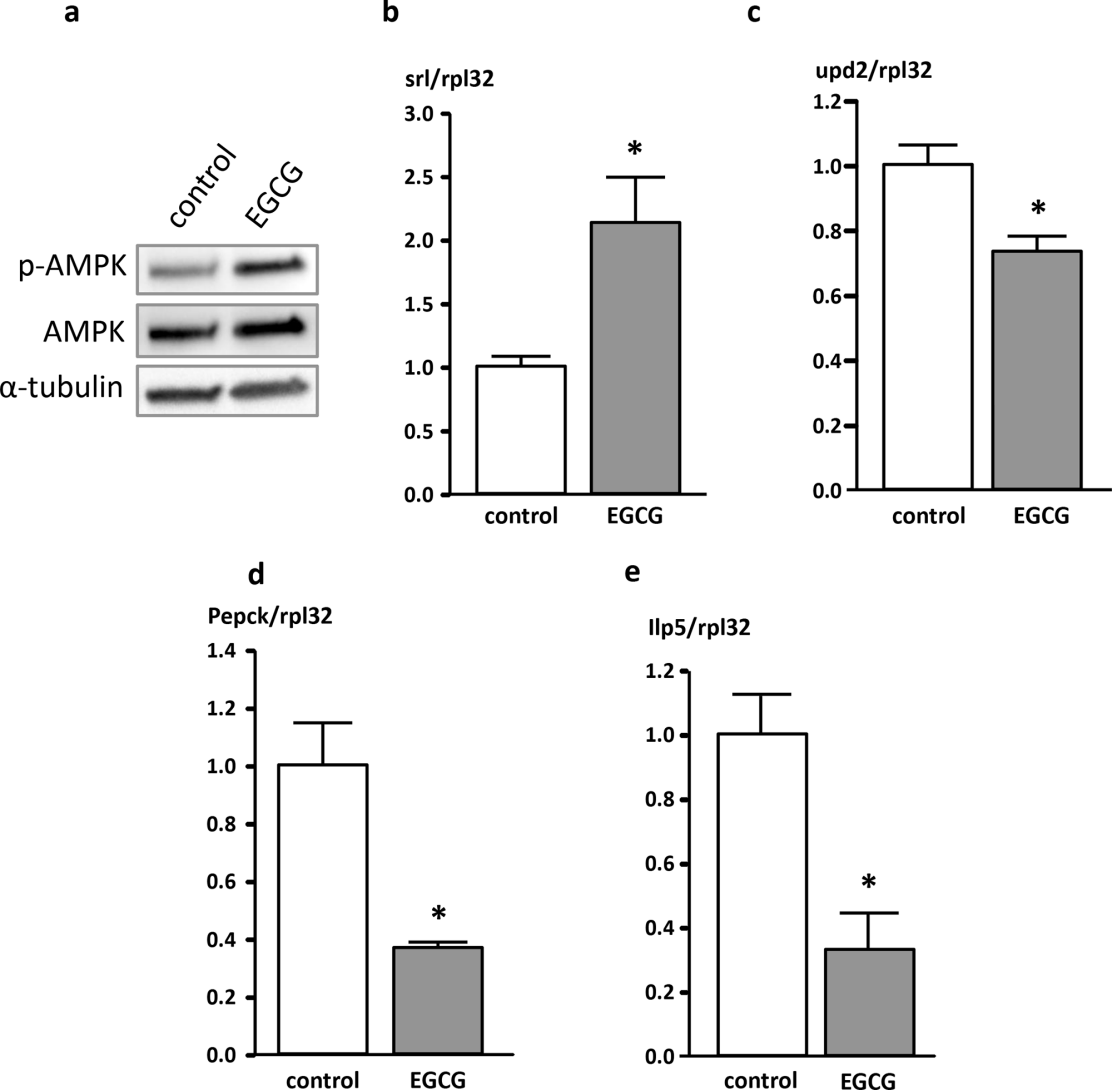
Protein (a) and mRNA (b-e) levels of genes involved in energy homeostasis in *Drosophila melanogaster* **a.** p-AMPK protein levels analyzed in whole fly homogenates following a 10 day treatment of male W^1118^ *Drosophila melanogaster* with 10 mg/ml EGCG. **b.** spargel (srl), **c.** unpaired 2 (upd2), **d.** phosphoenolpyruvate carboxykinase (Pepck), and **e.** insulin-like peptide 5 (Ilp5) mRNA levels of genes involved in energy homeostasis measured in whole fly homogenates after a 10 day supplementation period of male W^1118^ *Drosophila melanogaster* with 10 mg/ml EGCG. The data are expressed as the mean + SEM (n = 3, indicating extraction from 3 × 5 flies). * indicates significant differences in EGCG-treated flies compared to flies fed control medium for 10 days (p < 0.05, Student's t-test).

Flies treated with EGCG for 10 days exhibited higher levels of p-AMPK protein as depicted in Figure 4a. Furthermore, flies housed on an EGCG-supplemented diet showed significantly higher levels of spargel (srl) mRNA expression than the corresponding control flies (Figure 4b). Srl is the fly homolog of human PPARγ-co-activator 1α (PGC1α), the master regulator of energy homeostasis. The mRNA expression of unpaired 2 (upd2), the homolog of human leptin, was significantly lowered in flies that had received EGCG for 10 days compared to control animals (Figure 4c). In addition, the key enzyme of gluconeogenesis, phosphoenolpyruvate carboxykinase (Pepck; Figure 4d), and insulin-like peptide 5 (Ilp5; Figure 4e) were significantly downregulated following EGCG-treatment for 10 days in comparison to flies receiving CM. Furthermore, the expression of Ilp2, Ilp3, and Ilp6 as well as the expression of the glucose transporters Glut1, Glut3 and Glut4EF was not affected by EGCG treatment (data not shown).

## DISCUSSION

In the present study we used *Drosophila melanogaster* as a model organism in order to elucidate the effect of EGCG on glucose metabolism, fitness and lifespan. *Drosophila melanogaster* offers a wide variety of advantages. This includes a short generation time, a reasonable number of offspring and a relatively short lifespan. Furthermore, it possesses defined organs including gut, brain and fat body. *Drosophila melanogaster* holds a complex and dynamic gut exhibiting a similar structure and organization like the mammalian gut predestining it as a feasible model organism in nutrition research [32–35]. The application of the EGCG-rich green tea extract significantly extended lifespan in male flies. Similarly to our results indicating that EGCG only extended the lifespan in male but not in female flies (data not shown), Lopez and co-workers found that lifespan is only extended in male but not in female *Drosophila melanogaster* following EGCG application [13]. It has also been shown that in male flies a lifelong intake of 10 mg/ml green tea catechin extract extended the mean lifespan (59 ± 2.8 days) compared to the corresponding control flies (51 ± 2.0 days) [12].

We identified EGCG as an inhibitor of α-amylase and α-glucosidase, which may cause the attenuated glucose levels in our flies as the expression levels of the glucose transporters were not affected. We could also show via computational docking studies that EGCG fits into binding boxes of both α-amylase and α-glucosidase. In the case of α-amylase the docking results indicate competitive inhibition by EGCG through a single high-affinity binding site located close to the active center. In contrary docking with α-glucosidase rather suggests a noncompetitive inhibition mode with EGCG blocking the entry of the deeply buried substrate-binding site. Our results also demonstrate that the blocking effect of EGCG is stronger with regard to α-glucosidase than α-amylase. Interestingly, a recent study conducted by Harrison and co-workers identified the α-amylase inhibitor acarbose as a compound that extends lifespan in mice [36].

Tinkerhess and colleagues showed that, as in mammals, endurance exercise caused an upregulation of the PGC1α homolog srl in *Drosophila melanogaster*. Interestingly, in mutant srl flies, the positive effect of exercise is abolished whereas an overexpression of srl in the muscle and the heart of the mutant flies improved some exercise-related physiological parameters [37]. A 30 day application of the EGCG-rich tea extract to our flies resulted in improved climbing activity that may be related to the significant increase of the srl expression and consequently to a potential increase in mitochondrial biogenesis. These characteristics may also contribute to the extension in lifespan. The weakness of the current study is a missing experiment with srl mutant flies. Further studies are needed to investigate the effects of EGCG on srl mutants.

We were able to detect changes in the expression of parameters involved in energy metabolism. With regard to the insulin-like peptides (Ilps), Ilp5 was found to be significantly downregulated by EGCG in our *Drosophila melanogaster*. Min and co-workers also observed a downregulation of Ilp5 mRNA levels following dietary restriction [38, 39], and another group demonstrated that yeast dietary restriction (DR) (in the fly medium the percentage of yeast was decreased although the percentage of carbohydrate remained constant) caused a downregulation of both Ilp5 mRNA and protein levels [38, 40]. At the same time, the glucose levels in our EGCG-treated flies were significantly lower than in the corresponding control flies, which may explain the extension in lifespan by EGCG. Glucose has been described as a pro-aging factor that interferes with all relevant regulators of the aging process [41, 42]. This has also been reported by Schulz and co-workers who observed a decrease in lifespan of *Caenorhabditis elegans* due to high glucose availability while glucose restriction resulted in an extended lifespan through an induction of the mitochondrial respiration [43]. The downregulation of the rate-controlling enzyme in gluconeogenesis, Pepck, may have also contributed to the lower glucose levels in our EGCG-treated flies. This result is in contrast to the fact that an increase in PGC1α is related to an upregulation of gluconeogenesis including an induction of Pepck [44]. Indeed, our results are supported by two recent studies. Aatsinki and co-workers [45] showed an upregulation of hepatic PGC1α and a simultaneous impairment of hepatic Pepck by metformin treatment in both human primary hepatocytes and mice. Metformin is an anti-diabetic drug used clinically for decades and known for its AMPK-inducing and lifespan extending effects [46–49]. Doan et al. [50] treated mice with gallic acid (GA), a natural polyphenol, and observed a significant induction of AMPK activation accompanied by an increase in PGC1α and a decrease in Pepck expression. In *Caenorhabditis elegans* it has been shown that *aak-2*, a homolog of the mammalian AMPK, is essentially required to extend lifespan due to glucose restriction. Schulz and colleagues suggest that the glucose restriction increases the levels of reactive oxygen species resulting in an induction of the endogenous antioxidant machinery consequently leading to an improved stress resistance which the authors refer to as “mitohormesis” [43]. The inhibition of gluconeogenesis in the present study may be at least partly mediated through an induction of AMPK by EGCG [51]. These results may imply that an EGCG treatment mimics the effects of caloric restriction (CR). To date, CR is the only established method to extend health and lifespan in primates [52–54].

**Figure 5:**
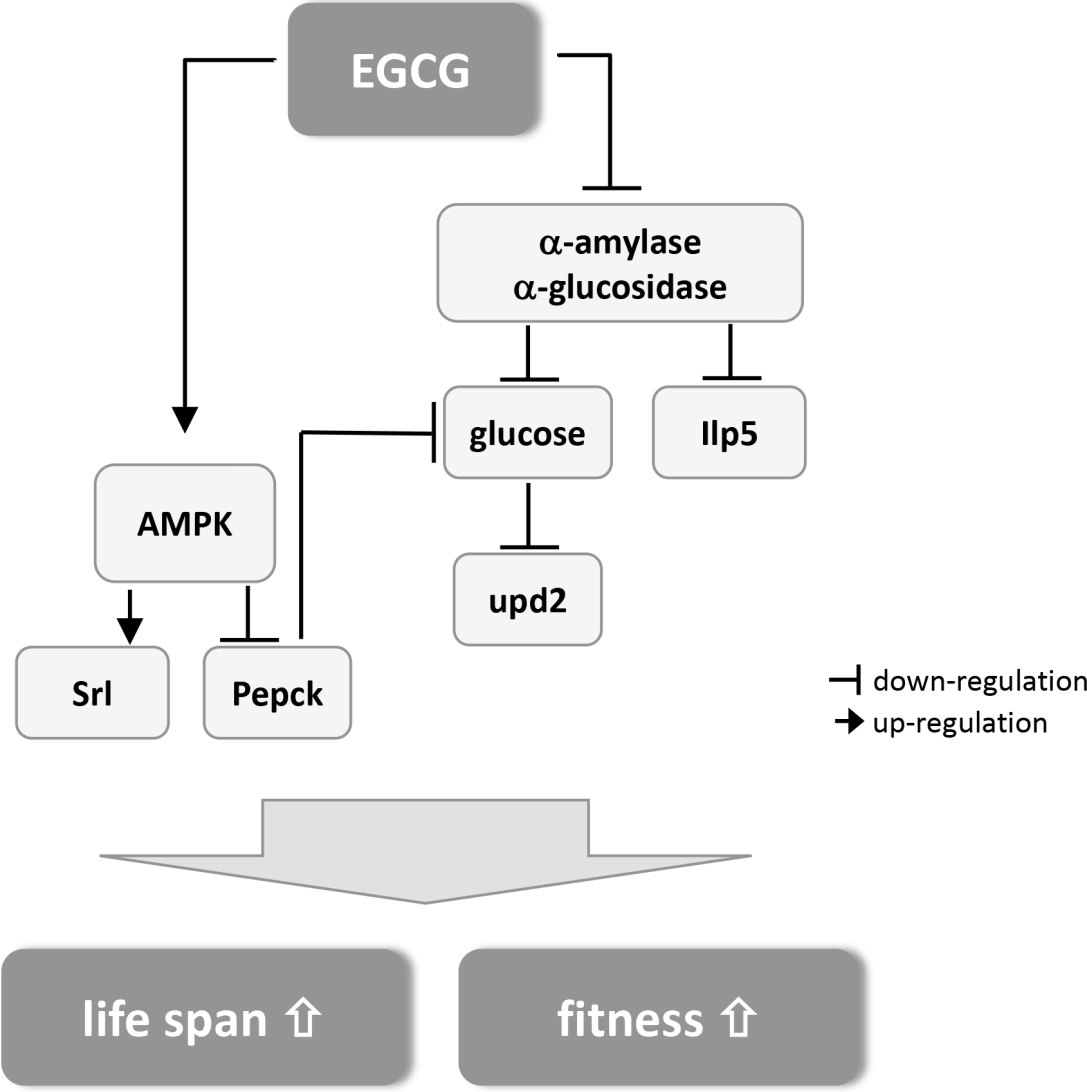
Schematic summary of the postulated mechanism how EGCG mediates lifespan extension and improved fitness in *Drosophila melanogaster* (for detailed description see text).

Our results indicate that EGCG significantly extended lifespan in male flies, which was accompanied by improved fitness. For the first time, we confirmed the inhibitory effect of EGCG on α-amylase and α-glucosidase activity *in vitro* and *in vivo* in *Drosophila melanogaster*. Computational docking analysis proved the potential of EGCG to dock into the substrate binding pocket of α-amylase and to a greater extent into α-glucosidase. By lowering the activity of these enzymes, EGCG causes a decrease in calorie uptake resulting in lower levels of glucose and Ilp5. The lowered energy intake also causes a significant downregulation of the leptin homolog upd2. The inhibition of the rate-limiting enzyme of gluconeogenesis, Pepck, is potentially mediated through an activation of AMPK by EGCG, which also causes the significant upregulation of srl, the master switch of mitochondrial biogenesis. The postulated mechanism is summarized in Figure 5.

Energy excess is the main cause of accelerated aging [55]. Limiting calorie intake may counteract the deleterious effects of aging [56]. We propose that EGCG may function as a potential caloric restriction mimetic, thereby affecting health and lifespan, which needs to be confirmed in future *in vivo* studies in mammalian species.

## MATERIALS AND METHODS

### *Drosophila melanogaster* stocks

In the present study W^1118^ wild-type *Drosophila melanogaster* were used for all experiments. Flies were maintained under conventional conditions on 10% Caltech (CT) medium (5.5% dextrose, 3.0% sucrose [both Carl Roth, Karlsruhe, Germany], 6.0% corn meal, 2.5% inactive dry yeast, 1.0% agar, 0.3% nipagin [all Dominique Dutscher SAS, Brumath, France] and 0.3% propionic acid [Carl Roth, Karlsruhe, Germany]) in a constant climate chamber (HPP 1018, Memmert, Schwabach, Germany) with a temperature of 25°C, 60% relative humidity and a 12-h day/night cycle. For all experiments, age-matched flies from synchronized eggs were used.

### Gustatory assay

In order to exclude differences in food intake as reason for differing results between control flies and tea polyphenol treated flies, the gustatory assay was performed using the method of Bahadorani et al. [18]. Fifteen flies were either kept on control medium (CM; 5% sucrose, 8.6% corn meal, 5% inactive dry yeast, 0.5% agar) or isocaloric CM supplemented with 10 mg/ml tea polyphenols (EGCG; Teavigo, DSM, Basel, Switzerland). They were reared under standard conditions (25°C, 60% relative humidity, 12-h day/night cycle), and the medium was changed at least 2 times during this period. After 5 days, flies were transferred onto CM or EGCG stained with 0.2% sulforhodamine B sodium salt (Sigma-Aldrich, Steinheim, Germany) and kept under standard conditions for 15 h. Flies were anesthetized and sorted to an arbitrary redness index of their abdomens (colorless = 0, partly colored = 0.5, fully red colored = 1.0). The food intake did not differ between control flies and EGCG-treated flies.

### Lifespan experiments

Twelve vials containing 20 male flies each were divided into two groups. One group (*n* = 120) received CM; the other group (*n* = 120) received the EGCG-supplemented diet. Flies were reared under standard conditions (25°C, 60% relative humidity, 12-h day/night cycle), and the medium was changed every 2–3 days. This procedure was adopted for all experiments of the study if not otherwise noted. Dead flies were counted every 2–3 days and analyzed for survivorship using dLife [19].

### Climbing assay

Climbing ability was assayed as an indicator of overall fitness of the flies. Flies were maintained under standard conditions and received either CM (*n* = 100) or EGCG (*n* = 100) for 30 days. On day 30, the flies were transferred into empty vials and the RING-assay described by Gargano et al. [20] was performed. In brief, vials with 10 flies each were placed in a box containing a metering rule. The vial-containing box was rapidly tapped three times on the table, and a photo was taken after 4 s of climbing time. This procedure was repeated ten times with a 30 second break between trials. Data analysis was performed using the method of Bazzell et al. [21] with modifications.

### Glucose analysis

Flies were maintained either on CM (*n* = 15) or EGCG (*n* = 15) under standard conditions for 10 days. Five flies were homogenized in 250 μl PBS plus 0.01% Triton^™^ X-100 (Sigma-Aldrich, Steinheim, Germany) using a Qiagen TissueLyser II (Hilden, Germany) at 4°C and 5000 × *g* for 10 min. Fly lysates were centrifuged at 5000 × *g* at 4°C for 10 min. The supernatant was removed and stored at −80°C until use. Glucose levels were detected by Fluitest^®^GLU (Analyticon Biotechnologies, Lichtenfels, Germany) according to the manufacturer's instructions. Sample concentrations were calculated via the standard curve and related to the corresponding fly weights per fly.

### *In vitro* α-amylase and α-glucosidase assay

The α-glucosidase and α-amylase inhibition assay was performed using the method of Phan et al. [22] with modifications. To test α-glucosidase inhibition, 50 μl of test sample/H_2_O were mixed with 100 mmol/l KH_2_PO_4_ (pH = 6.8) and 50 μl α-glucosidase (0.5 U/ml, Sigma-Aldrich, Steinheim, Germany) and incubated at 37°C for 5 min. One unit of enzyme activity is defined as the amount of enzyme that is needed for the liberation of 1 μmol of *p*-nitrophenol from *p*-nitrophenyl-α-D-glucopyranoside (*p*NPG). Subsequently, 50 μl of 10 mmol/l *p*NPG was added, mixed and incubated at 37°C for 20 min. The reaction was stopped by adding 2 mol/l Na_2_CO_3_. The absorbance of the liberated *p*-nitrophenol was measured at 405 nm in a plate reader (Tecan, Crailsheim, Germany). To test for α-amylase inhibition, 50 μl of test sample/H_2_O were mixed with 50 μl 1% starch solution and 50 μl α-amylase (10 U/ml, Sigma-Aldrich, Steinheim, Germany) and incubated at 20°C for 3 min. Subsequently, 50 μl of color reagent (44 mM 3,5-Dinitrosalicylic Acid Solution (DNS) with 1.1 M Sodium Potassium Tartrate Solution) and 50 μl α-amylase (as a control) were added, mixed, spun down and incubated at 99°C for 15 min. After cooling, 450 μl H_2_O was added, and the absorbance was measured at 540 nm in a plate reader (Tecan, Crailsheim, Germany). The % inhibition of enzyme activity was calculated by the following formula ((Δ H_2_O - Δ sample)/Δ H_2_O)*100; Δ = absorbance test group (including enzyme) - absorbance control group (without enzyme). Acarbose was used as positive control. Acarbose and EGCG were dissolved in H_2_O.

### *In vivo* α-glucosidase and amylase activity assay

Flies were maintained either on CM (*n* = 39) or EGCG (*n* = 39) under standard conditions for 10 days. Per group, 13 male flies were homogenized in 250 μl Ca^2+^/Mg^2+^-free PBS (Gibco, Darmstadt, Germany) using a Qiagen TissueLyser II (Hilden, Germany). Homogenates were centrifuged at 13,000 × *g* at 4°C for 10 min. Supernatants were removed and used for enzyme activity measurements.

The α-glucosidase activity assay (MAK123, Sigma-Aldrich, Steinheim, Germany) was performed according to the manufacturer's instructions.

The amylase activity assay (MAK009, Sigma-Aldrich, Steinheim, Germany) was conducted according to the manufacturer's instructions. Enzyme activities were related to the corresponding fly weights per fly.

### Docking analysis

X-ray structural models of human salivary α-amylase (PDB ID: 1SMD [23]), isomaltase from *Saccharomyces cerevisiae* (PDB ID: 3A4A [24]) and pig pancreatic α-amylase (PDB ID: 1PPI [25]) were obtained from the Protein Data Bank at Brookhaven. The EGCG 3D-structure was obtained from the Zinc database (EGCG: Zinc ID 3870412) [26]. The human salivary α-amylase structure was prepared for docking by removing from the crystallographic structure ions and water molecules. Since the salivary R-amylase X-ray structure does not contain hydrogen atoms, H-atoms were added to the enzyme prior to docking by the SwissDock web-service [27]. (The web service SwissDock is based on fast docking using the CHARMM force field with the docking software EADock DSS [28]). For structure homology modeling the sequence of α-glucosidase (gi|411229) from baker's yeast and the high resolution X-ray structure of isomaltase (PDB ID: 3A4A [24]) were submitted to the web-based service of SwissModel [29] along previously reported methods [30]. The obtained target-template sequence similarity (SID = 72.3) and the global model quality estimation (GMQE = 0.92) were judged to be suitable for docking experiments. Molecular analyses were performed with the UCSF Chimera package. Chimera is developed by the Resource for Biocomputing, Visualization, and Informatics at the University of California, San Francisco (supported by NIGMS P41-GM103311) [31].

### RNA isolation and real time PCR

Total RNA was isolated from 5 flies per sample using peqGOLD TriFast^™^ (Peqlab, Erlangen, Germany) according to the manufacturer's protocol. RNA concentration was determined by measuring the absorbance with a NanoDrop^®^ spectrophotometer (Thermo Scientific, Langenselbold, Germany), and the RNA purity was assessed by calculating the 260/280-nm and 260/230-nm ratios. RNA aliquots were stored at −80°C until the PCR was performed. The primers for *Drosophila melanogaster* genes were designed using Primer3 software and were synthesized and purchased from MWG Biotech, Ebersberg, Germany (see Table 1). The real time PCR was performed using the SensiFast™ SYBR^®^ No-ROX One-Step kit (Quantace, Berlin, Germany) on a Rotor-Gene 6000 cycler (Corbett Life Science, Sydney, Australia). The relative mRNA levels of the target genes were calculated relative to the expression of the housekeeping gene ribosomal protein l32 (rpl32).

**Table 1:** Primer sequences (*Drosophila melanogaster*) used for real time PCR

gene	forward primer (5′ → ‘3)	reverse primer (3′ → ‘5)	PCRproduct (bp)	annealing temperature (°C)
**Ilp5**	tgatggacatgctgagggtt	catgtggtgagattcggagc	128	57
**Pepck**	ccgccgagaaccttattgtg	agaatcaacatgtgctcggc	136	57
**rpl32**	ggcaagcttcaagatgacca	gttcgatcctaaccgatgt	198	58
**spargel**	ctcttggagtccgagatccgcaa	gggaccgcgagctgatggtt	90	64
**upd2**	atgatcctgagcgtcgtgat	cccgatgatgaggatgacga	131	59

Abbreviations: Ilp5 = insulin-like peptide 5; Pepck = phosphoenolpyruvate carboxykinase; rpl32 = ribosomal protein l32; upd2 = unpaired 2; bp = base pairs

### Western blotting

Per group, 5 flies were homogenized in RIPA buffer (50 mmol/l Tris, 150 mmol/l NaCl, 0.5% sodium deoxycholate (v/v), 0.1% SDS (w/v) and 1% NP-40 (v/v), at pH 7.4) supplemented with phosphatase and proteinase inhibitors in a Qiagen TissueLyser II (Hilden, Germany). Samples were incubated on ice for 30 min and centrifuged at 13,000 × *g* at 4°C for 20 min. The supernatant was aliquoted and stored at −80°C until analysis. Protein concentrations were detected using the Pierce^™^ BCA protein assay kit (Darmstadt, Germany) according to the manufacturer's instructions. A quantity of 50 μg of each sample was heated with loading buffer and separated on a 4–20% ready-to-use gel (Bio-Rad, Munich, Germany). Then, samples were transferred onto a polyvinylidenedifluoride membrane (Bio-Rad, Munich, Germany) and blocked with 5% (w/v) skim milk dissolved in Tris-buffered saline + 0.05% (v/v) Tween 20 for 2 h. Membranes were probed overnight with p-AMPK (#2535, 1:1000, Cell Signaling, Frankfurt, Germany), AMPK (#ab80039, 1:1000, Abcam, Cambridge, UK) and α-tubulin (#2125, 1:1000 Cell Signaling, Frankfurt, Germany) primary antibodies followed by incubation with the corresponding secondary antibodies, anti-rabbit (1:4000, Bio-Rad, Munich, Germany) for p-AMPK and α-tubulin and anti-mouse (1:4000, Bio-Rad, Munich, Germany) for AMPK, at room temperature for 1 h. Bands were visualized with ECL substrate (Thermo Fisher Scientific, Schwerte, Germany) in a ChemiDoc XRS System (Bio-Rad, Munich, Germany) using Quantity One Software (version 4.6.3; Bio-Rad, Munich, Germany).

### Statistics

Analysis of lifespan and stress test experiments was performed with dLife (freeware, available from the laboratory of Scott Pletcher, University of Michigan, Ann Arbor, MI, USA). Survival was calculated using a Kaplan-Meier approach, and significant differences were calculated applying the log-rank test with the dLife program based on R. Mean and median survival times were calculated with SPSS (Statistical Package for the Social Sciences, IBM, Armonk, NY, USA). All other data were analyzed for significant differences using a one-way ANOVA followed by a Student's *t*-test. All data were tested for normality of distribution (Kolmogorov-Smirnov and Shapiro-Wilk) and homogeneity of variances (Levene's test). *P* < 0.05 was considered significant. The data are expressed as the mean ± SEM.
